# Regional variation in length of stay for stroke inpatient rehabilitation in traditional Medicare and Medicare Advantage

**DOI:** 10.1093/haschl/qxae089

**Published:** 2024-07-16

**Authors:** Dian Luo, Mariétou H Ouayogodé, John Mullahy, Ying (Jessica) Cao

**Affiliations:** Department of Population Health Sciences, University of Wisconsin–Madison, Madison, WI 53726, United States; Department of Population Health Sciences, University of Wisconsin–Madison, Madison, WI 53726, United States; Center for Demography and Health of Aging, University of Wisconsin–Madison, Madison, WI 53726, United States; Department of Population Health Sciences, University of Wisconsin–Madison, Madison, WI 53726, United States; Center for Demography and Health of Aging, University of Wisconsin–Madison, Madison, WI 53726, United States; Department of Population Health Sciences, University of Wisconsin–Madison, Madison, WI 53726, United States; Center for Demography and Health of Aging, University of Wisconsin–Madison, Madison, WI 53726, United States; Health Innovation Program, University of Wisconsin–Madison, Madison, WI 53726, United States

**Keywords:** traditional Medicare, Medicare Advantage, inpatient rehabilitation, regional variation

## Abstract

Regional variation in health care use threatens efficient and equitable resource allocation. Within the Medicare program, variation in care delivery may differ between centrally administered traditional Medicare (TM) and privately managed Medicare Advantage (MA) plans, which rely on different strategies to control care utilization. As MA enrollment grows, it is particularly important for program design and long-term health care equity to understand regional variation between TM and MA plans. This study examined regional variation in length of stay (LOS) for stroke inpatient rehabilitation between TM and MA plans in 2019 and how that changed in 2020, the first year of the COVID-19 pandemic. Results showed that MA plans had larger across-region variations than TM (SD = 0.26 vs 0.24 days; 11% relative difference). In 2020, across-region variation for MA further increased, but the trend for TM stayed relatively stable. Market competition among all inpatient rehabilitation facilities (IRFs) within a region was associated with a moderate increase in within-region variation of LOS (elasticity = 0.46). Policies reducing administrative variation across MA plans or increasing regional market competition among IRFs can mitigate regional variation in health care use.

## Introduction

Substantial regional variations in Medicare spending and use have been documented over the past several decades.^1-4^ Unexplained regional variations in health care spending and use are interpreted as an indicator of wasteful spending in higher spending regions and as an indicator of suboptimal levels of service in regions with lower use. These unwanted regional variations represent a threat to the equitable and efficient allocation of health care resources. On the one hand, studies showed that variation in health care spending and use was not explained by differences in the frequency and severity of health conditions across regions but rather by differences in clinical practice patterns and health care institutions.^1-3^ On the other hand, studies also found very weak associations between higher health care spending and better quality of care or care outcomes.^2,5^

Post-acute care (PAC) has been shown to be the major driver of regional variation in Medicare expenditure, accounting for over three-quarters of the total variation in the traditional Medicare program (TM) since 2010.^2,6^ Studies on regional variations in PAC have focused predominantly on skilled-nursing facilities and home health (as these 2 accounted for almost 80% of PAC spending in 2021),^3,4^ but gave very limited attention to inpatient rehabilitation facilities (IRFs). However, IRFs offer the most intensive PAC, have the highest per-episode cost and cost growth,^7,8^ as well as the widest range of per-episode cost across the country.^9,10^ These facts make IRFs a target with a large potential to reduce regional variations in PAC spending and in Medicare spending overall.

Previous studies on regional variation in health care use have also focused largely on TM with less evidence on Medicare Advantage (MA) plans.^3,4^ In 2023, MA plan enrollment reached 50% out of all Medicare beneficiaries and is expected to be the major Medicare coverage type in the next decade and after.^8,11^ Research has shown that beneficiaries with MA plans use less care and have lower costs, but no worse care outcomes than TM beneficiaries. Many believe these differences are due to the fact that MA plans are private managed-care plans with capitated payment models, and hence, bear more financial risks and are more incentivized to control care use and care costs.^12-15^ However, the regional variation in health care use for MA plans relative to TM is not known. Medicare Advantage plans may help reduce regional variation to the same extent as they reduce inappropriate care.^16,17^ Alternatively, MA plans could have larger regional variation than TM due to private and more segmented managed care, which makes such plans more sensitive to local professional norms, clinical practice patterns, or health care institutions than the centrally administered TM.^13,18,19^

With the unexpected public health shock of the COVID-19 pandemic, TM and MA plans could differ from each other even more in terms of regional variation since the 2 coverage types rely on different administrative procedures to monitor and adjust care use.^20-22^ Research has shown that non–COVID-19 care utilization significantly decreased in all care sectors, including inpatient care, since the outbreak of the COVID-19 pandemic.^23,24^ Studies also showed that patients and providers shortened length of stay (LOS) for hospitalizations^23,24^ and/or used PAC as a substitute to minimize the risk of infections and to open up resources to take care of patients with COVID-19.^21,22^ Hospital discharge patterns across PAC facilities also changed, with significantly more discharges to home with home health care, slightly more discharges to IRFs (when more intensive therapies were needed for continuous recovery), and significantly fewer to skilled-nursing facilities.^21,22,25-27^ In addition to these across-the-board changes, the differences in pandemic timing and severity across states and regions, as well as the local market structures such as MA penetration and IRF competition, may interact with care operations, contributing to more complicated changes in regional variation between TM and MA plans.

This study assessed the regional variation in LOS for stroke patients admitted to IRFs between TM and MA plans during the COVID-19 pandemic in 2020 relative to the previous year. The study also identified specific regions and regional factors that drive or shape the variations in the 2 Medicare coverage types over time. Our focus on stroke patients is motivated by the fact that stroke is the most common admission condition for the aging population who use PAC in IRFs.^28,29^ Stroke also requires nonelective care, such that the variation in care utilization should depend proportionally more on medical needs and clinical reasons such as severity, the (specifically) affected brain regions and relevant comorbidities,^30,31^ and relatively less on local institutions or care practice patterns than other admission conditions. Our focus on the variation in LOS as the primary outcome is supported by the fact that LOS is a direct measure of care utilization that is roughly proportional to care costs in the IRF settings.^7,9^ Using LOS instead of care costs (reimbursement or payment) can help isolate the effect of price and payment differences across geographic regions and obtain a clearer assessment of regional variation and comparison in care utilization between TM and MA plans.

## Data and methods

### Study design

A retrospective multiyear, cross-sectional study design was used to investigate the variation across 10 Centers for Medicare and Medicaid (CMS) administrative regions^32^ in LOS for Medicare beneficiaries who were admitted to IRFs for stroke in 2019 and 2020. Comparisons in regional variation were made between 2 Medicare coverage types—TM and MA—as well as between 2 periods defined by the admission date: the pre-pandemic period, which was defined as from January 1, 2019, to February 29, 2020, and during the pandemic period, from March 1, 2020, to December 31, 2020.

### Data source

We used the Inpatient Rehabilitation Facility—Patient Assessment Instrument (IRF-PAI) data provided by the Uniform Data System for Medical Rehabilitation (UDSMR) for analysis. The UDSMR maintains the world's largest government-independent repository for rehabilitation outcomes and IRF-PAI data.^33^ The IRF-PAI is required by the CMS for reimbursement purposes.^34^ By CMS rules, IRF-PAI reports patient demographic characteristics, pre-admission and postdischarge locations, patient-episode medical conditions (eg, case-mix group [CMG],^35^ comorbidity tiers), IRF characteristics, and cost factors such as LOS, payment amount, primary and secondary payment sources (eg, TM and MA), etc.^36,37^ The IRF-PAI also includes patients’ functionality, which is assessed within 72 hours after admission and before discharge, and information on therapy receipt, care interruption, and other critical practice details.^37^

Medicare beneficiaries who were 65 years or older at the time of admission were included in the study. The study sample excluded a patient-episode if the patient was not admitted for initial rehabilitation (*n* = 4601), if the pre-hospital living setting was non-home (*n* = 2710), if the patient died during the rehabilitation stay (*n* = 1610), if the LOS was longer than 30 days (*n* = 2915) or shorter than 3 days (*n* = 2489), and if the patient experienced program interruption (*n* = 1887) or was discharged against medical advice (*n* = 385). Indicators of these exclusion criteria were obtained directly from IRF-PAI questionnaires. Patient-episodes with missing information on critical variables of interest (admission date, payer/coverage type, LOS, etc) were also excluded (*n* = 2945) (Appendix A1). The inclusion and exclusion criteria followed existing studies using the same dataset.^15,28,29,38^

To address the potential sample selection issues that could arise at the stages of Medicare plan selection, hospitalization, admission to an IRF, as well as between the pre-pandemic and during-pandemic periods, 2 rounds of propensity score matching were conducted to obtain a comparable and balanced sample.^20^ In the first round, we used 1-to-1 exact matching based on patient demographics, medical conditions, and IRF characteristics to obtain TM and MA matched episode pairs. This round was conducted separately for pre-pandemic and during-pandemic periods. In the second round, we matched TM and MA pairs from the first round between pre-pandemic and during-pandemic periods to ensure the matched pairs were comparable over 2 periods.

### Outcome and exposure variables of interest

The primary outcome of this study was the regional variation in LOS among stroke patients discharged from IRFs for the 10 CMS administrative regions.^32^ Two measures of variation were calculated: within- and across-region variations. Within-region variation was calculated as the standard deviation of risk-adjusted LOS for all patient-episodes within a CMS region. It measured the variation of LOS among all episodes of service delivered within the region after accounting for patient and facility characteristics. Across-region variation was calculated as the weighted standard deviation of the average of risk-adjusted LOS for the 10 CMS regions. The weight equaled the admission volume of each region. Across-region variation measured the nationwide variation in LOS across the 10 CMS regions.

This study chose to use broader CMS regions rather than more granular geographic categorization, such as hospital referral regions (HRRs), as in other studies for hospital inpatient care because there were only 1152 IRFs in 2019 (vs 6120 hospitals). With the relatively unequal geographic distribution of IRFs (compared to hospitals) across the United States, the use of HRRs (*n* = 304) as the geographic unit will result in many places having no or very few IRFs and hence being excluded from the analysis. Even categorizing by state (*n* = 50) will yield some states having no or fewer than 3 IRFs for a certain facility type (in-hospital or freestanding unit). In order to maintain the national scope for analysis and also to pass the data-use requirement for minimum cell counts, CMS regions were used.

The exposure variables of interest were the Medicare coverage type and the pandemic timing. The Medicare coverage type (ie, TM and MA plans) was used to compare regional variations in LOS between the 2 major payment models. The pandemic timing (ie, the pre-pandemic and during-pandemic periods) was used to compare the regional variation in LOS over time and the differences in regional variations between the 2 coverage types over time.

### Explanatory variables

Patient demographics, patient-episode–level medical conditions, and IRF facility characteristics were used as explanatory variables in the patient-episode–level analysis. Patient demographics included age, sex, race/ethnicity (non-Hispanic White, Black or Hispanic, Asian or other), marital status (married or living with a partner, single, other or unknown), and Medicare and Medicaid dual coverage. Medical conditions included CMG and comorbidity tier (Tier). The CMG and Tier are the 2 primary factors that determine IRF reimbursement and the suggested LOS for each admission according to CMS Prospective Payment Systems (PPS).^39^ There are 6 CMGs for stroke (ie, 101–106), indicating different severity and affected brain regions. There are 4 comorbidity tiers (ie, none, minor, moderate, and major) that can pair with each CMG. The CMG and Tier can jointly absorb the variations of LOS due to medical severity and need. Facility characteristics included IRF facility type (in-hospital or freestanding unit) and number of beds. In addition, CMS region and admission-month fixed effects were also included as control variables. Inclusion of explanatory variables was based on data availability and existing literature in the same field.^15,28,29,38^

Two region-level characteristics were computed for each of the 10 CMS regions, both before and during the pandemic—the Herfindahl-Hirschman index (HHI) and MA penetration—and were used as explanatory variables in the region-level analysis. The HHI is a measure of market concentration of all IRFs operating within a region. It is defined as $\mathrm{HHI}=\sum_{i=1}^{K}S_{i}^{2}$, where *S_i_* is the market share of IRF *i* in a CMS region and *K* is the total number of IRFs in that region. In this study, market share of IRF *i* is defined as the percentage (0%–100%) of episodes delivered by IRF *i* out of all episodes delivered by all *K* IRFs in a region. Intuitively, HHI is proportional to the average market share, weighted by market share. As such, it can range from 0 to 1.0, where 0 means perfect competition and 1 means monopoly. Increases in the HHI generally indicate a decrease in competition and an increase in market power or market concentration. The US Department of Justice considers a market with an HHI of less than 0.15 to be competitive, an HHI between 0.15 and 0.25 to be moderately concentrated, and an HHI of 0.25 or greater to be highly concentrated.^40,41^ Medicare Advantage penetration is calculated as the market share (0%–100%) of IRF episodes delivered to MA beneficiaries out of all episodes delivered in a region.^42,43^ These 2 measures captured the market structure at the regional level.

### Analytical approach

First, in the patient-episode–level analysis, a Poisson regression model was used to predict the risk-adjusted LOS by regressing the actual LOS on pandemic timing, Medicare coverage type, patient demographic characteristics, medical conditions, IRF facility characteristics, region, and month fixed effects. Two-way and 3-way interaction terms between CMS region, pandemic timing, and Medicare coverage type were also included in the model. The average risk-adjusted LOS and the (within-region) standard deviation of risk-adjusted LOS were calculated by CMS region, pandemic timing, and Medicare coverage type using the Margin command in postestimation procedure in STATA software (version 17, StataCorp, College Station, TX).

Next, for across-region analysis, we ranked the average risk-adjusted LOS across the 10 CMS regions from the shortest to longest by pandemic timing and Medicare coverage type to show the relative changing ranges and rank order. The standard deviation of risk-adjusted LOS across CMS regions was calculated and tested for difference between the 2 pandemic timing periods and the 2 Medicare coverage types. In order to show the relative changes among regions before and during the pandemic and compare between the 2 coverage types, maps of all CMS regions with their estimated average risk-adjusted LOS were generated.

Last, to analyze how macro–health care market structure affected the regional average risk-adjusted LOS and the within-region variation in LOS, log-log form regression models were used to regress the logarithm of the regional average of LOS (or standard deviation of LOS) on the logarithm of HHI (or MA penetration). The estimated coefficient from the log-log model is proportional to the ratio of percentage change in the outcome variable to the percentage change in the explanatory variable, representing elasticity. Elasticity is an economic concept that measures the sensitivity of (or change in) an outcome (eg, quantity demand) in response to the change in a variable of interest (eg, price). In the IRF context, it can be interpreted as the percent changes in average LOS (or standard deviation of LOS) to every 1% change in HHI (or MA penetration). The values of the estimated elasticities imply the sensitivity of the outcomes of interest to changes in the market structure.

For the sensitivity analysis, we stratified the study sample by patient CMG and repeated the above analyses by each CMG. Stratifying patients by CMG yielded more homogeneous patient-episode subsamples in terms of medical conditions, which allowed for more refined estimation. We used different time points to define the pandemic timing, such as by discharge date, admission date, and at the beginning of March, mid-March, or the beginning of April 2020. We also used the unmatched full sample for analysis to compare the results from using a matched and balanced sample. All regression analyses were conducted using STATA software (version 17). Figures and maps were generated using R software (version 4.2.1; R Foundation for Statistical Computing, Vienna, Austria).

### Limitations

This study has a few limitations. First, the study focused on Medicare beneficiaries who were 65 years or older at the time of IRF admission. The results may not apply to a younger population. Second, the study used the 10 CMS administrative regions to categorize geographic areas based on data availability. A more granular categorization, such as state, county or HRR, may produce different results. However, using more granular categorization will result in the exclusion of some geographic areas and loss of the national scope in the analysis due to the fact that many HRRs or counties have no or very few IRFs and some states have no or fewer than 3 IRFs for a certain facility type (in-hospital or freestanding unit). Third, this study used stroke as our admission condition of interest. The study's implications may not be generalizable to other conditions, such as hip fracture or joint replacement. Nonetheless, stroke is an acute care, nonelective condition for which medical conditions, such as severity, affected brain regions, and comorbidities, explain proportionally more variation in care utilization than other nonmedical factors, including local institutions or care practice patterns. As a result, using stroke as a case study to analyze social and institutional regional variation after adjusting for necessary clinical and medical risk factors can provide a benchmark for other IRF admission conditions. Fourth, the study period lasted only 2 years. A longer observation period may help retrieve more consistent long-term trends. Fifth, results from the study suggested only an association rather than causality. One cannot test possible mechanisms that contribute to the differences between TM and MA plans. Last, this study used regional variation in care utilization (ie, LOS) as 1 aspect of health care inequity. While LOS can provide valuable insights, it may not fully isolate the effects of price and payment differences across regions without considering other relevant factors. There are also many other aspects of health care inequities, not measured in our study, including health care resources and provider distribution^44^ and environmental and/or socioeconomic structural determinants that may be explored in future studies.

## Results

### Characteristics of the study sample

The final study sample included 72 794 patient-episodes after matching, 36 448 of which were covered by TM and 36 346 of which were covered by MA. The study sample had an average age of 76 years, 51% female, 76% non-Hispanic White, 47% married or living with a partner, and 9% with Medicare and Medicaid dual coverage. Forty-seven percent of patient-episodes had no comorbidity, 48% had minor comorbidities, and the remaining patient-episodes had major or moderate comorbidities. Distribution of these patient-episodes across the 6 CMGs ranged from 5% (CMG 101) to 31% (CMG 106). Approximately 42% of all episodes were delivered by freestanding IRFs. The average number of certified beds for all IRFs was 48. The distribution of patient-episodes across the 10 CMS regions as well as of the above-mentioned characteristics was comparable between TM and MA and before and during the pandemic period (Appendix A2).

### Average LOS and variation across regions

On average, episodes covered by MA plans had significantly longer LOS (15.22 days; 95% CI: 15.16, 15.27) than those covered by TM (15.02 days; 95% CI: 14.97, 15.08; difference: 0.19 days; 95% CI: 0.11, 0.27) before the pandemic. During the pandemic, average LOS for both coverage types significantly decreased after accounting for patient and IRF facility characteristics. The average LOSs covered by TM and MA plans were not significantly different from each other (TM: 14.85 days; 95% CI: 14.80, 14.91; MA: 14.83 days; 95% CI: 14.78, 14.89) (Table 1).

**Table 1.** Risk-adjusted length of stay across 10 Centers for Medicare and Medicaid Services regions, 2019–2020.

	Traditional Medicare				Medicare Advantage			
	Pre-pandemic	Rank	During pandemic	Rank	Pre-pandemic	Rank	During pandemic	Rank
Region 1	15.37 (15.10, 15.64)	8	14.82 (14.57, 15.08)	5	15.42 (15.16, 15.68)	8	15.12 (14.86, 15.39)	9
Region 2	15.12 (14.91, 15.34)	6	15.30 (15.08, 15.51)	10	14.85 (14.65, 15.04)	2	14.32 (14.12, 14.51)	1
Region 3	14.69 (14.53, 14.86)	1	14.72 (14.56, 14.88)	3	15.18 (15.01, 15.34)	3	14.98 (14.81, 15.15)	6
Region 4	14.96 (14.84, 15.07)	4	14.67 (14.55, 14.78)	2	15.24 (15.12, 15.36)	4	14.68 (14.56, 14.80)	3
Region 5	14.92 (14.78, 15.06)	3	14.62 (14.48, 14.76)	1	15.39 (15.25, 15.54)	7	14.82 (14.68, 14.96)	4
Region 6	15.21 (15.07, 15.35)	7	15.00 (14.86, 15.14)	6	15.30 (15.15, 15.44)	6	14.95 (14.81, 15.10)	5
Region 7	15.43 (15.18, 15.69)	9	15.28 (15.03, 15.53)	8	15.54 (15.30, 15.79)	9	15.57 (15.32, 15.82)	10
Region 8	15.67 (15.33, 16.02)	10	15.18 (14.85, 15.51)	7	15.73 (15.40, 16.06)	10	15.01 (14.68, 15.34)	7
Region 9	14.76 (14.57, 14.94)	2	14.77 (14.59, 14.95)	4	14.61 (14.43, 14.79)	1	14.57 (14.39, 14.75)	2
Region 10	15.06 (14.75, 15.37)	5	15.28 (14.97, 15.59)	9	15.26 (14.94, 15.57)	5	15.04 (14.73, 15.36)	8
Average LOS across regions	15.02 (14.97, 15.08)		14.85 (14.80, 14.91)		15.22 (15.16, 15.27)		14.83 (14.78, 14.89)	
Difference during vs pre-pandemic			−0.17 (−0.25, −0.09)***				−0.38 (−0.46, −0.30)***	
Difference, MA vs TM					0.19 (0.11, 0.27)***		−0.02 (−0.10, 0.06)	
Variation/SD across regions	0.235		0.238		0.261		0.271	
Difference during vs pre-pandemic			*P* < .16				*P* < .05	
Difference, MA vs TM					*P* < .05		*P* < .05	

Abbreviations: LOS, length of stay; MA, Medicare Advantage; TM, traditional Medicare.

Source: Authors’ analysis of the Inpatient Rehabilitation Facility—Patient Assessment Instrument (IRF-PAI) data, 2019–2020.

Notes: 95% CIs are shown in parentheses. The *P* value of the SD difference is based on the Bartlett's equal variances test. Ranks 1–10 represent the shortest to the longest LOS. ***Stand for 99%, 95% and 90% significant level.

**Figure 1.**
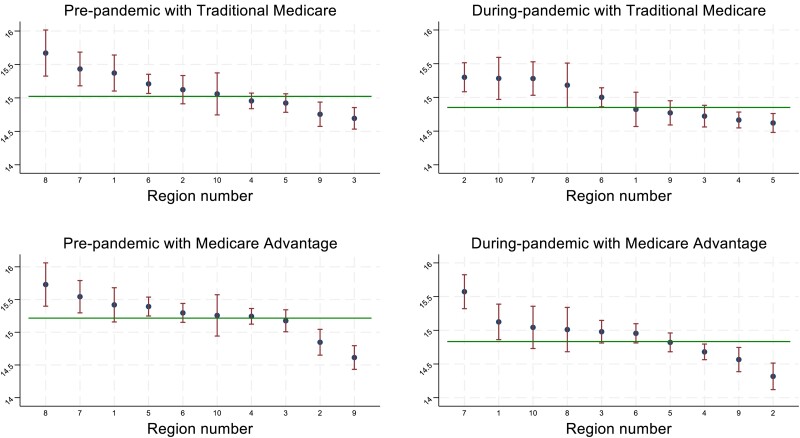
Average length of stay (in days) across regions between traditional Medicare and Medicare Advantage, 2019–2020. Source: Authors’ analysis of the Inpatient Rehabilitation Facility—Patient Assessment Instrument (IRF-PAI) data, 2019–2020. Each graph shows the average length of stay with 95% CIs in each region. The horizontal reference line represents the average length of stay across regions in each graph.

The variation in (average) LOS across the 10 CMS regions was also larger for MA-covered episodes than for TM-covered episodes (SD: MA vs TM, 0.261 vs 0.235; *P* = .03) before the pandemic. During the pandemic, across-region variation in LOS increased for MA-covered episodes (SD: 0.271 vs 0.261; *P* = .04) but stayed almost unchanged for TM-covered episodes (SD: 0.238 vs 0.235; *P* = .16), yielding even larger differences in across-region variation between the 2 coverage types (SD: 0.271 vs 0.238; *P* = .02) (Table 1). Figure 1 shows the trends in LOS variations across regions by the pandemic timing and the Medicare coverage type. For both average LOS and variation in LOS, the differences between TM and MA before and during the pandemic were clinically and economically important based on the relative percentage changes and the large population who used the care services.^15,20^

**Figure 2.**
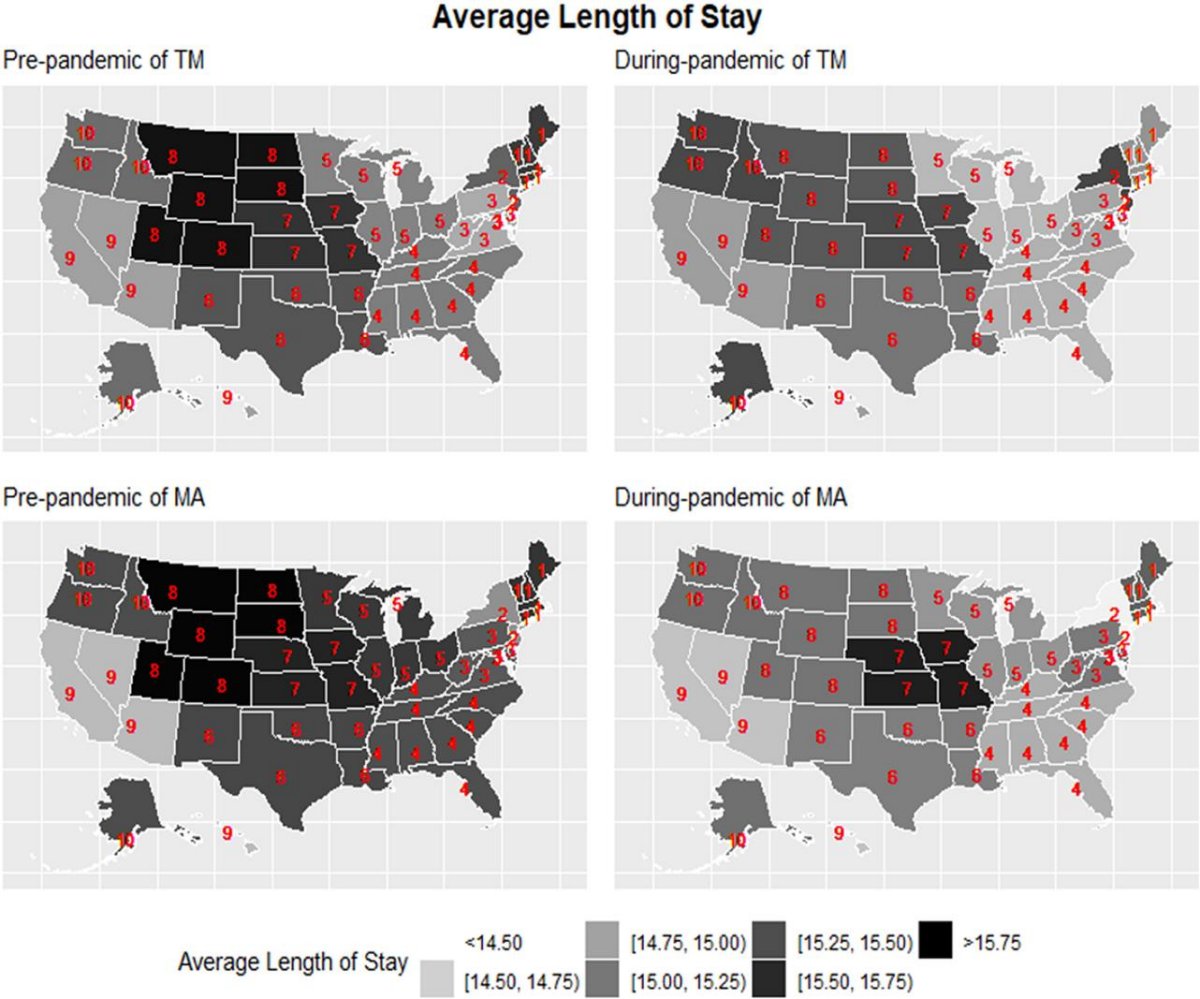
Map of average length of stay (in days) across the Centers for Medicare and Medicaid Services (CMS) regions, 2019–2020. Source: Authors’ analysis of the Inpatient Rehabilitation Facility—Patient Assessment Instrument (IRF-PAI) data, 2019–2020. Red numbers identify the 10 CMS administrative regions. Abbreviations: MA, Medicare Advantage; TM, traditional Medicare.

Ranking the risk-adjusted average LOS of the 10 CMS regions from shortest to longest (1 to 10, where 1 means the shortest LOS) and displaying them in maps, results suggested that TM-covered episodes showed a mild self-correction towards the average LOS across regions, but MA-covered episodes showed slightly greater deviations away from the average. For TM-covered episodes, Regions 8, 7, and 1 had a historically longer LOS than the national average and regions 3 and 9 had a shorter LOS. All of these regions improved their relative rankings by moving towards the national average during the pandemic. In contrast, for MA-covered episodes, regions with historically longer (Regions 7 and 1) and shorter (Regions 9 and 2) LOS turned out to be even more polarized in the relative ranking across regions during the pandemic (Figures 1 and 2).

Sensitivity analysis of the stratified study sample for each CMG suggested consistent findings on across-region variations. Compared with TM-covered episodes, MA-covered episodes had either larger across-region variations before the pandemic or more increases (or fewer decreases) in the variation during the pandemic or both, yielding significantly higher across-region variations by the end of the study period. The only exception was CMG 104, for which TM-covered episodes showed much larger increases in across-region variations during the pandemic than MA-covered episodes (Appendixes A3 and A4). Sensitivity analysis of using alternative definitions on the pandemic timing and using the unmatched national sample also yielded consistent findings.

### Regional factors influencing within-region variation

The HHIs of IRFs within each CMS region ranged between 0.01 and 0.07 and averaged 0.028 (SD = 0.02). The values, being well below 0.15, implied that the regional markets were highly competitive, with an average of 18–20 IRFs in each state and 80–100 IRFs in each region. During the pandemic, there was no significant changes in HHI or market competitiveness relative to pre-pandemic levels. The HHI had almost negligible effects on the average LOS within a region, but had moderately large effects on the within-region variation in LOS. The estimated elasticity suggested that, for every 1% increase in the value of HHI (average, 0.028), the within-region standard deviation of LOS increased by 0.458% (95% CI: 0.456%, 0.459%) for TM and by 0.455% (95% CI: 0.453%, 0.456%) for MA before the pandemic. The elasticity of within-region variation on HHI slightly increased for MA during the pandemic (0.459; 95% CI: 0.457, 0.460) but stayed almost unchanged for TM. In general, a higher HHI or market concentration (lower competition) among IRFs increased the variation in LOS for that region. The sensitivity (elasticity) of the variation in LOS to market concentration was lower for MA than for TM before the pandemic, but became higher for MA than for TM during the pandemic. However, the differences in elasticity were not statistically significant between TM and MA before or during the pandemic (Table 2 and Appendix A5).

**Table 2.** Elasticity of length of stay (LOS) and variation in LOS on market concentration, 2019–2020.

	Pre-pandemic		During pandemic	
	TM	MA	TM	MA
Elasticity of LOS on HHI	0.007***	0.004***	0.010***	0.010***
	(0.007, 0.008)	(0.004, 0.004)	(0.010, 0.010)	(0.010, 0.011)
Elasticity of SD of LOS on HHI	0.458***	0.455***	0.458***	0.459***
	(0.456, 0.459)	(0.453, 0.456)	(0.457, 0.460)	(0.457, 0.460)
Elasticity of LOS on MA penetration	−0.016***	−0.024***	−0.012***	−0.012***
	(−0.017, −0.016)	(−0.025, −0.024)	(−0.012, −0.011)	(−0.012, −0.011)
Elasticity of SD of LOS on MA penetration	−0.013**	−0.024***	−0.022***	−0.022***
	(−0.024, −0.002)	(−0.035, −0.013)	(−0.035, −0.009)	(−0.035, −0.009)
Mean risk-adjusted LOS (SD)	15.120 (0.308)	15.251 (0.323)	14.965 (0.276)	14.907 (0.342)
Mean risk-adjusted SD of LOS (SD)	0.109 (0.040)	0.108 (0.038)	0.107 (0.038)	0.107 (0.038)
Mean HHI (SD)	0.028 (0.020)		0.028 (0.020)	
Mean MA penetration (SD)	0.194 (0.052)		0.250 (0.050)	

Abbreviations: HHI, Herfindahl-Hirschman index; MA, Medicare Advantage; TM, traditional Medicare; SD, standard deviation.

Source: Authors’ analysis of the Inpatient Rehabilitation Facility—Patient Assessment Instrument (IRF-PAI) data, 2019–2020.

Notes: 95% CIs are shown in parentheses unless otherwise noted. *** and ** stand for 99%, 95% and 90% significant level.

Medicare Advantage penetration for the CMS regions ranged between 15% and 35% and averaged 19.4% (SD = 0.05%) before the pandemic and 25.4% (SD = 0.05%) during the pandemic. Medicare Advantage penetration had a significantly negative association with both the average LOS and the (within-region) variation in LOS, but the magnitude of the estimates was very small. Estimated elasticity suggested that every 1% increase in MA penetration was associated with an average or standard deviation of LOS decrease by only 0.01% to 0.02% (Table 2, Appendix A6).

## Discussion

Using the US national patient functional assessment data, this study examined the regional variation in LOS for Medicare beneficiaries admitted for stroke in IRF settings. Comparisons were made between patient-episodes that were covered by TM and MA plans during the first year of the COVID-19 pandemic (2020) relative to the previous year. The study also assessed specific regions and regional factors that contributed to regional variation and their changes over time. Findings from the study generated the following points that are worth a discussion.

This study found that, while the average risk-adjusted LOS decreased during the pandemic for both TM and MA plans, changes in regional variation in LOS between these 2 Medicare coverage types were different. Relative to TM, regional variation in LOS for MA plans was higher in the pre-pandemic period and increased even more during the pandemic. In contrast, regional LOS for TM was relatively stable over time. The findings of reduced LOS during the pandemic are consistent with the literature, showing that patients decreased care utilization overall,^23,45^ shortened hospital LOS,^24^ reduced use of PAC,^22^ used home-based care to substitute for institutional care,^25-27^ or reduced LOS in IRF settings during that period.^20^ Our findings on larger and increasing regional variation in LOS for MA plans relative to TM have important implications. With the general concerns of health care equity and efficiency,^1,2,5^ and the fact that MA accounted for more than half of all Medicare beneficiaries since 2023,^9,11^ further investigation into the sources and factors that are driving regional variation as well as the potential policies that can mitigate the unwanted variation are needed.^3,6,18,46^

The average risk-adjusted LOS by CMS region and the relative ranking changes provide some additional insights on the larger regional variation in MA relative to TM over time. For TM, the regions with historically longer and shorter LOS converged towards the national average during the pandemic. In contrast, for MA plans, the regions with longer or shorter LOS became even more polarized over time. One possible reason for these different changes between TM and MA plans is that TM relies on a centrally administered PPS^39^ to guide the care delivery and monitor the reimbursement process, whereas MA plans are individually managed by various commercial insurers in their region of operation. Under PPS, IRF payments for patient-episodes are predetermined by patients’ CMG and comorbidity tier (Tier) at admission.^35,39^ The CMS also provides a menu of suggested LOSs for all CMG-Tier combinations, which is adjusted annually based on the past 5-year moving average of all episodes delivered for the same CMG-Tier combination nationwide.^47,48^ To the extent that PPS regulates LOS and mitigates the unwanted variation across regions over time, CMS payment policies have the potential to improve geospatial health care equity.^3,49^

Last, the study examined IRF market concentration and MA penetration as 2 regional factors that could influence the within-region variation in LOS. Results suggested that IRF market concentration measured by HHI had a moderately large positive association with the within-region variation in LOS. Every 1% increase in HHI was associated with a within-region variation (measured by standard deviation) increase of 0.46%, indicating within-region variation elasticity (sensitivity) to market concentration. In addition to the existing literature that showed the association between market competition and care intensity (or care utilization) among the institutional providers,^40,41^ this study found that market structure may also influence the variations in care intensity or utilization (eg, LOS per episode) within the local markets. Proper regulation of market concentration could also help improve health care equity.

## Conclusion

This study examined the risk-adjusted average LOS and the regional variation in LOS between TM and MA beneficiaries in IRFs over time. Results suggest an increasing regional variation for MA plans and a relatively stable and slowly self-correcting regional variation for TM. The universal administration of TM by CMS might be a possible explanation. Further, proper market regulation and some increase in market competition could help reduce regional variations and improve geospatial health care equity.

## Supplementary Material

qxae089_Supplementary_Data
