# Supplementary material for: Regional variation in length of stay for stroke inpatient rehabilitation in traditional Medicare and Medicare Advantage
Source: Health Aff Sch. 2024 Jul 16;2(7):qxae089. doi: 10.1093/haschl/qxae089 (PMC11282463; doi:10.1093/haschl/qxae089)
Supplement: qxae089_Supplementary_Data [file qxae089_supplementary_data.zip › Appendix_LOS RV TM MA_formatted.pdf]

Appendix A1 (Figure). Study Sample Flow Chart

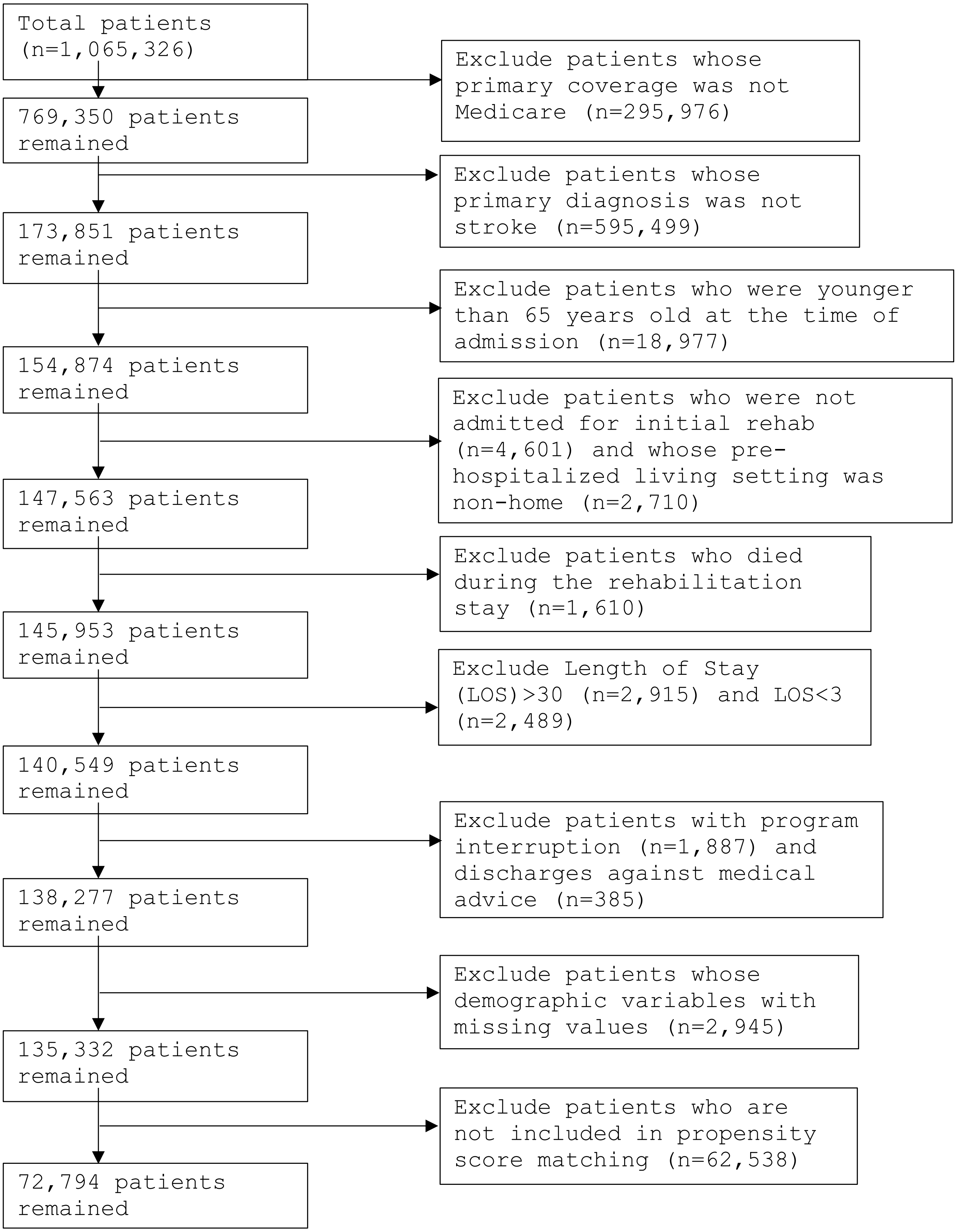

Appendix A2 (Table). Demographic Characteristics of the Study Sample

	Traditional Medicare N= 36,448				Medicare Advantage N= 36,346			
	Pre-Pandemic N= 18,249		During N= 18,199		Pre-Pandemic N= 18,148		During N= 18,198	
	**N**	**%**	**N**	**%**	**N**	**%**	**N**	**%**
**Age (Mean/SD)**	76.38	0.05	76.48	0.05	76.47	0.05	76.39	0.05
**Gender**								
Male	8,955	49.07	8,844	48.6	8,711	48	8,790	48.3
Female	9,294	50.93	9,355	51.4	9,437	52	9,408	51.7
**Race**								
White	13,555	74.28	13,526	74.32	13,386	73.76	13,407	73.67
Black or Hispanic	4,178	22.89	4,132	22.7	4,252	23.43	4,236	23.28
Asian or Other	516	2.83	541	2.97	510	2.81	555	3.05
**Marital Status**								
Married	8,667	47.49	8,503	46.72	8,518	46.94	8,557	47.02
Single	8,862	48.56	8,931	49.07	8,904	49.06	8,920	49.02
Other/Unknown	720	3.95	765	4.2	726	4	721	3.96
**Dual Coverage**								
No	16,575	90.83	16,470	90.5	16,450	90.64	16,475	90.53
Yes	1,674	9.17	1,729	9.5	1,698	9.36	1,723	9.47
**CMG**								
101	821	4.5	866	4.76	870	4.79	828	4.55
102	2,247	12.31	2,326	12.78	2,277	12.55	2,312	12.7
103	4,853	26.59	4,720	25.94	4,720	26.01	4,770	26.21
104	3,213	17.61	3,239	17.8	3,226	17.78	3,256	17.89
105	1,376	7.54	1,372	7.54	1,343	7.4	1,331	7.31
106	5,739	31.45	5,676	31.19	5,712	31.47	5,701	31.33
**Tier**								
None	8,737	47.88	8,680	47.69	8,652	47.67	8,628	47.41
Major	352	1.93	382	2.1	398	2.19	381	2.09
Medium	242	1.33	254	1.4	261	1.44	265	1.46

Minor	8,918	48.87	8,883	48.81	8,837	48.69	8,924	49.04
**IRF Facility type**								
Freestanding	7,507	41.14	7,635	41.95	7,568	41.7	7,541	41.44
Hospital In-Unit	10,742	58.86	10,564	58.05	10,580	58.3	10,657	58.56
**CMS Region**								
Region 01	853	4.67	915	5.03	888	4.89	876	4.81
Region 02	1,298	7.11	1,260	6.92	1,456	8.02	1,411	7.75
Region 03	2,160	11.84	2,186	12.01	2,052	11.31	2,061	11.33
Region 04	4,172	22.86	4,047	22.24	4,078	22.47	4,142	22.76
Region 05	3,085	16.91	2,936	16.13	2,955	16.28	3,014	16.56
Region 06	2,814	15.42	2,861	15.72	2,790	15.37	2,771	15.23
Region 07	947	5.19	997	5.48	986	5.43	978	5.37
Region 08	543	2.98	563	3.09	578	3.18	562	3.09
Region 09	1,744	9.56	1,781	9.79	1,724	9.5	1,732	9.52
Region 10	633	3.47	653	3.59	641	3.53	651	3.58

Source: Authors' Analysis of the Inpatient Rehabilitation Facility - Patient Assessment Instrument (IRF-PAI) data 2019-2020.

Notes: Acronyms: CMG-case-mixed group; IRF-inpatient rehabilitation facilities; CMS-Centers for Medicare and Medicaid Services.

The table shows the count and percentage of each covariate used in our margin estimation except for age. The age is described by mean and sd.

Appendix A3 (Table). Pooled Regression with Full Risk Adjustment Across Regions by Case-Mixed Group (CMG)

**CMG101**	**Traditional Medicare (TM) (95% CI)**				**Medicare Advantage (MA)**			
	**Pre-Pandemic**		**During Pandemic**		**Pre-Pandemic**		**During Pandemic**	
Region 1	7.52	(6.66, 8.38)	7.52	(6.70, 8.34)	8.42	(7.66, 9.17)	7.09	(6.35, 7.83)
Region 2	8.97	(8.09, 9.84)	7.99	(7.13, 8.84)	8.84	(8.03, 9.64)	8.51	(7.65, 9.37)
Region 3	8.06	(7.45, 8.67)	7.70	(7.10, 8.31)	7.93	(7.31, 8.55)	7.35	(6.79, 7.90)
Region 4	8.02	(7.57, 8.48)	7.33	(6.92, 7.74)	8.40	(7.96, 8.83)	8.07	(7.63, 8.51)
Region 5	8.15	(7.69, 8.60)	7.93	(7.53, 8.34)	8.01	(7.57, 8.45)	7.51	(7.05, 7.97)
Region 6	8.53	(7.98, 9.08)	7.56	(7.05, 8.07)	7.76	(7.23, 8.29)	7.69	(7.15, 8.22)
Region 7	7.99	(7.21, 8.77)	7.88	(7.18, 8.59)	8.75	(8.01, 9.48)	7.82	(7.09, 8.55)
Region 8	8.83	(7.99, 9.66)	7.81	(6.87, 8.75)	8.33	(7.52, 9.13)	8.21	(7.36, 9.06)
Region 9	7.52	(6.91, 8.13)	7.76	(7.16, 8.37)	7.84	(7.18, 8.49)	7.93	(7.32, 8.54)
Region 10	7.86	(7.12, 8.60)	8.02	(7.27, 8.77)	7.24	(6.52, 7.97)	7.92	(7.17, 8.67)
Average	8.12	(7.93, 8.32)	7.71	(7.52, 7.89)	8.11	(7.92, 8.30)	7.78	(7.59, 7.97)
LOS Difference								
Pre vs. During-pandemic	-0.42	(-0.69, -0.14)			-0.34	(-0.61, -0.07)		
TM vs. MA	-0.01	(-0.28, 0.27)	0.07	(-0.20, 0.34)				
Standard Deviation (SD)	0.385		0.235		0.381		0.356	
P-value of SD Difference								
Pre vs. During-pandemic	<0.05				<0.05			
TM vs. MA	0.15		<0.05					

**CMG102**	**Traditional Medicare (TM) (95% CI)**				**Medicare Advantage (MA)**			
	**Pre-Pandemic**		**During Pandemic**		**Pre-Pandemic**		**During Pandemic**	
Region 1	10.27	(9.73, 10.82)	10.20	(9.64, 10.76)	10.46	(9.89, 11.02)	10.09	(9.59, 10.60)
Region 2	11.21	(10.62, 11.79)	10.65	(10.11, 11.19)	10.84	(10.33, 11.35)	9.50	(9.00, 10.01)
Region 3	9.92	(9.53, 10.32)	9.70	(9.33, 10.07)	10.15	(9.75, 10.56)	10.16	(9.76, 10.56)

Region 4	10.44	(10.13, 10.75)	9.45	(9.17, 9.73)	10.51	(10.21, 10.81)	10.11	(9.83, 10.39)
Region 5	10.01	(9.73, 10.30)	9.73	(9.42, 10.04)	10.15	(9.85, 10.44)	9.87	(9.57, 10.18)
Region 6	10.51	(10.13, 10.89)	9.80	(9.45, 10.15)	10.48	(10.12, 10.84)	9.97	(9.61, 10.34)
Region 7	10.63	(10.10, 11.17)	10.05	(9.57, 10.53)	10.81	(10.23, 11.40)	11.06	(10.53, 11.60)
Region 8	10.39	(9.68, 11.10)	9.86	(9.22, 10.50)	10.38	(9.73, 11.02)	10.28	(9.65, 10.91)
Region 9	10.10	(9.67, 10.53)	9.94	(9.53, 10.35)	9.86	(9.39, 10.32)	9.50	(9.04, 9.96)
Region 10	9.84	(9.27, 10.42)	9.67	(9.10, 10.25)	10.52	(9.94, 11.10)	9.49	(8.99, 10.00)
Average	10.30	(10.16, 10.43)	9.80	(9.68, 9.93)	10.37	(10.24, 10.51)	10.00	(9.87, 10.12)
LOS Difference								
Pre vs. During Pandemic	-0.49	(-0.68, -0.31)			-0.38	(-0.56, -0.19)		
TM vs. MA	0.08	(-0.11, 0.26)	0.19	(0.01, 0.37)				
Standard Deviation (SD)	0.342		0.306		0.273		0.351	
P-value of SD Difference								
Pre vs. During Pandemic	<0.05				<0.05			
TM vs. MA	<0.05		<0.05					

**CMG103**	**Traditional Medicare (TM) (95% CI)**				**Medicare Advantage (MA)**			
	**Pre-Pandemic**		**During Pandemic**		**Pre-Pandemic**		**During Pandemic**	
Region 1	12.99	(12.53, 13.45)	12.22	(11.79, 12.65)	13.67	(13.19, 14.15)	12.75	(12.28, 13.22)
Region 2	12.49	(12.13, 12.84)	12.76	(12.38, 13.14)	13.74	(13.34, 14.14)	12.67	(12.30, 13.03)
Region 3	12.65	(12.36, 12.94)	12.28	(12.00, 12.57)	13.27	(12.97, 13.58)	12.83	(12.54, 13.13)
Region 4	12.64	(12.43, 12.86)	12.44	(12.22, 12.66)	12.93	(12.72, 13.15)	12.48	(12.27, 12.70)
Region 5	12.80	(12.56, 13.04)	12.14	(11.90, 12.38)	13.16	(12.91, 13.41)	12.33	(12.09, 12.56)
Region 6	12.74	(12.47, 13.01)	12.38	(12.11, 12.65)	12.47	(12.20, 12.73)	12.08	(11.81, 12.35)
Region 7	13.32	(12.86, 13.78)	12.59	(12.17, 13.02)	12.43	(11.98, 12.88)	12.47	(12.06, 12.87)
Region 8	12.93	(12.36, 13.50)	12.19	(11.63, 12.75)	13.19	(12.59, 13.79)	12.46	(11.87, 13.05)
Region 9	12.45	(12.12, 12.77)	12.25	(11.93, 12.57)	11.91	(11.59, 12.22)	12.02	(11.70, 12.34)
Region 10	12.62	(12.09, 13.15)	12.86	(12.37, 13.36)	12.47	(11.97, 12.97)	12.60	(12.09, 13.10)

Average	12.72	(12.62, 12.82)	12.37	(12.27, 12.47)	12.91	(12.81, 13.01)	12.43	(12.33, 12.53)
LOS Difference								
Pre vs. During Pandemic	-0.34	(-0.49, -0.20)			-0.48	(-0.63, -0.34)		
TM vs. MA	0.19	(0.05, 0.34)	0.05	(-0.09, 0.20)				
Standard Deviation (SD)	0.194		0.190		0.507		0.256	
P-value of SD Difference								
Pre vs. During Pandemic	<0.05				<0.05			
TM vs MA	<0.05		<0.05					

**CMG104**	**Traditional Medicare (TM) (95% CI)**				**Medicare Advantage (MA)**			
	**Pre-Pandemic**		**During Pandemic**		**Pre-Pandemic**		**During Pandemic**	
Region 1	15.91	(15.25, 16.57)	15.08	(14.52, 15.64)	16.54	(15.88, 17.21)	16.21	(15.57, 16.86)
Region 2	16.17	(15.67, 16.67)	16.42	(15.91, 16.92)	16.35	(15.85, 16.84)	15.67	(15.19, 16.15)
Region 3	15.85	(15.47, 16.24)	15.35	(14.98, 15.72)	15.98	(15.57, 16.40)	15.84	(15.44, 16.23)
Region 4	15.90	(15.61, 16.19)	14.99	(14.71, 15.27)	16.15	(15.87, 16.44)	15.34	(15.06, 15.62)
Region 5	15.82	(15.46, 16.17)	15.41	(15.06, 15.76)	16.19	(15.83, 16.56)	15.83	(15.48, 16.17)
Region 6	16.13	(15.78, 16.48)	15.67	(15.31, 16.02)	16.42	(16.06, 16.78)	15.66	(15.31, 16.01)
Region 7	15.86	(15.21, 16.52)	16.07	(15.46, 16.68)	16.60	(15.98, 17.21)	16.42	(15.79, 17.06)
Region 8	16.82	(15.93, 17.70)	16.16	(15.30, 17.02)	16.10	(15.23, 16.96)	15.40	(14.60, 16.21)
Region 9	15.35	(14.93, 15.76)	14.95	(14.53, 15.38)	15.36	(14.94, 15.77)	15.24	(14.83, 15.66)
Region 10	15.30	(14.55, 16.04)	16.07	(15.29, 16.85)	16.57	(15.81, 17.34)	16.11	(15.35, 16.87)
Average	15.88	(15.74, 16.02)	15.43	(15.30, 15.57)	16.16	(16.03, 16.30)	15.66	(15.53, 15.80)
LOS Difference								
Pre vs. During Pandemic	-0.45	(-0.64, -0.26)			-0.50	(-0.70, -0.31)		
TM vs. MA	0.28	(0.09, 0.48)	0.23	(0.04, 0.42)				
Standard Deviation (SD)	0.286		0.450		0.319		0.322	

P-value of SD Difference								
Pre vs. During	<0.05				0.23			
TM vs. MA	<0.05		<0.05					

**CMG105**	**Traditional Medicare (TM) (95% CI)**				**Medicare Advantage (MA)**			
	**Pre-Pandemic**		**During Pandemic**		**Pre-Pandemic**		**During Pandemic**	
Region 1	17.29	(16.22, 18.35)	16.95	(15.99, 17.91)	17.69	(16.68, 18.70)	17.98	(17.07, 18.89)
Region 2	16.95	(16.16, 17.74)	16.92	(16.11, 17.74)	16.18	(15.43, 16.93)	16.29	(15.45, 17.13)
Region 3	16.17	(15.58, 16.77)	17.03	(16.43, 17.62)	17.38	(16.82, 17.95)	17.65	(17.06, 18.24)
Region 4	17.23	(16.76, 17.70)	16.99	(16.53, 17.46)	17.15	(16.65, 17.66)	17.19	(16.69, 17.70)
Region 5	16.85	(16.33, 17.38)	16.46	(15.92, 17.01)	17.94	(17.36, 18.53)	17.17	(16.62, 17.73)
Region 6	17.31	(16.77, 17.85)	16.98	(16.45, 17.51)	17.16	(16.58, 17.75)	17.22	(16.65, 17.79)
Region 7	16.28	(15.36, 17.21)	17.03	(16.01, 18.05)	17.50	(16.59, 18.41)	18.46	(17.46, 19.46)
Region 8	19.24	(17.94, 20.55)	18.75	(17.14, 20.35)	17.74	(16.43, 19.05)	17.82	(16.50, 19.14)
Region 9	16.97	(16.21, 17.73)	17.65	(16.94, 18.36)	16.72	(16.05, 17.39)	16.90	(16.22, 17.58)
Region 10	18.80	(17.29, 20.32)	17.81	(16.34, 19.28)	19.11	(17.50, 20.71)	16.63	(15.17, 18.09)
Average	17.03	(16.81, 17.25)	17.04	(16.82, 17.26)	17.31	(17.08, 17.53)	17.28	(17.05, 17.50)
LOS Difference								
Pre vs. During Pandemic	0.01	(-0.30, 0.32)			-0.03	(-0.34, 0.29)		
TM vs. MA	0.28	(-0.04, 0.59)	0.24	(-0.07, 0.55)				
Standard Deviation (SD)	0.632		0.451		0.582		0.481	
P-value of SD Difference								
Pre vs. During	<0.05				<0.05			
TM vs. MA	<0.05		<0.05					

**CMG106**	**Traditional Medicare (TM) (95% CI)**				**Medicare Advantage (MA)**			
	**Pre-Pandemic**		**During Pandemic**		**Pre-Pandemic**		**During Pandemic**	
Region 1	19.86	(19.28, 20.44)	19.50	(18.92, 20.07)	18.73	(18.20, 19.25)	19.00	(18.41, 19.59)
Region 2	18.88	(18.44, 19.32)	19.31	(18.88, 19.74)	17.52	(17.16, 17.89)	17.34	(16.97, 17.71)

Region 3	18.27	(17.95, 18.60)	18.88	(18.55, 19.22)	18.84	(18.50, 19.18)	18.59	(18.24, 18.94)
Region 4	18.75	(18.53, 18.97)	18.91	(18.68, 19.14)	19.10	(18.87, 19.34)	18.43	(18.21, 18.65)
Region 5	18.67	(18.38, 18.95)	18.77	(18.47, 19.06)	19.38	(19.07, 19.68)	18.91	(18.62, 19.20)
Region 6	19.19	(18.91, 19.46)	19.44	(19.17, 19.70)	19.54	(19.27, 19.82)	19.36	(19.09, 19.63)
Region 7	19.70	(19.20, 20.21)	20.05	(19.51, 20.59)	19.91	(19.42, 20.41)	20.05	(19.50, 20.60)
Region 8	19.66	(18.89, 20.43)	19.68	(19.01, 20.35)	20.35	(19.67, 21.04)	19.18	(18.47, 19.89)
Region 9	18.76	(18.39, 19.13)	19.16	(18.78, 19.53)	18.91	(18.53, 19.28)	18.73	(18.37, 19.10)
Region 10	19.63	(18.91, 20.35)	19.96	(19.23, 20.69)	19.42	(18.67, 20.18)	19.87	(19.08, 20.66)
Average	18.91	(18.80, 19.02)	19.16		19.12	(19.00, 19.23)	18.79	(18.67, 18.90)
LOS Difference								
Pre vs. During Pandemic	0.25	(0.09, 0.41)			-0.33	(-0.49, -0.17)		
TM vs. MA	0.20	(0.04, 0.36)	-0.38	(-0.54. -0.22)				
Standard Deviation (SD)	0.434		0.377		0.591		0.618	
P-value of SD Difference								
Pre vs. During	<0.05				<0.05			
TM vs. MA	<0.05		<0.05					

Source: Authors' Analysis of the Inpatient Rehabilitation Facility - Patient Assessment Instrument (IRF-PAI) data 2019-2020.
Notes: The P-value of standard deviation (SD) difference is based on the Bartlett's equal-variances test.

Appendix A4 (Figure). Average Length of Stay Across Case-Mixed Groups (CMG) Between Traditional Medicare and Medicare Advantage

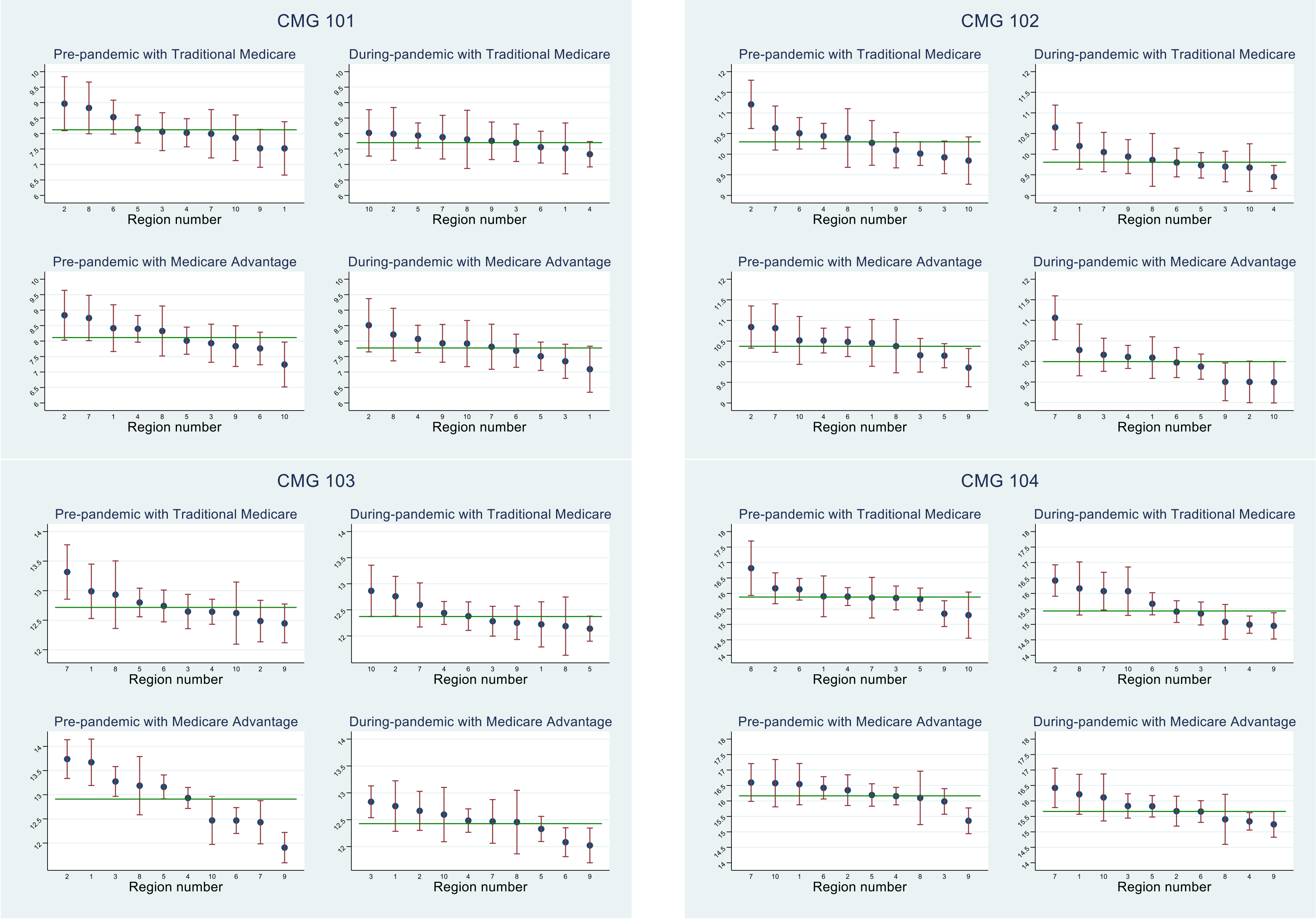

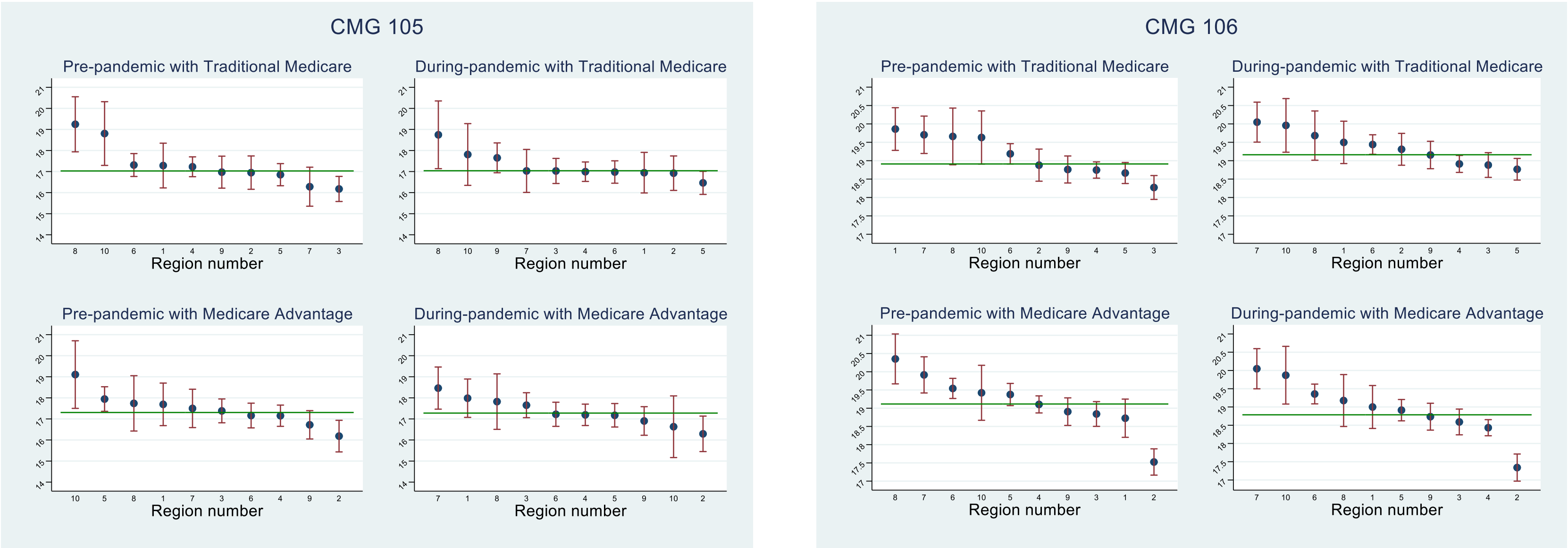

Source: Authors' Analysis of the Inpatient Rehabilitation Facility - Patient Assessment Instrument (IRF-PAI) data 2019-2020.

Notes: Each graph shows the average length of stay with 95% CI in each region. The green line represents the average length of stay across regions in each graph.

Appendix A5 (Figure). Association of Market Concentration with Risk-Adjusted Length of Stay (LOS) and Variation of LOS

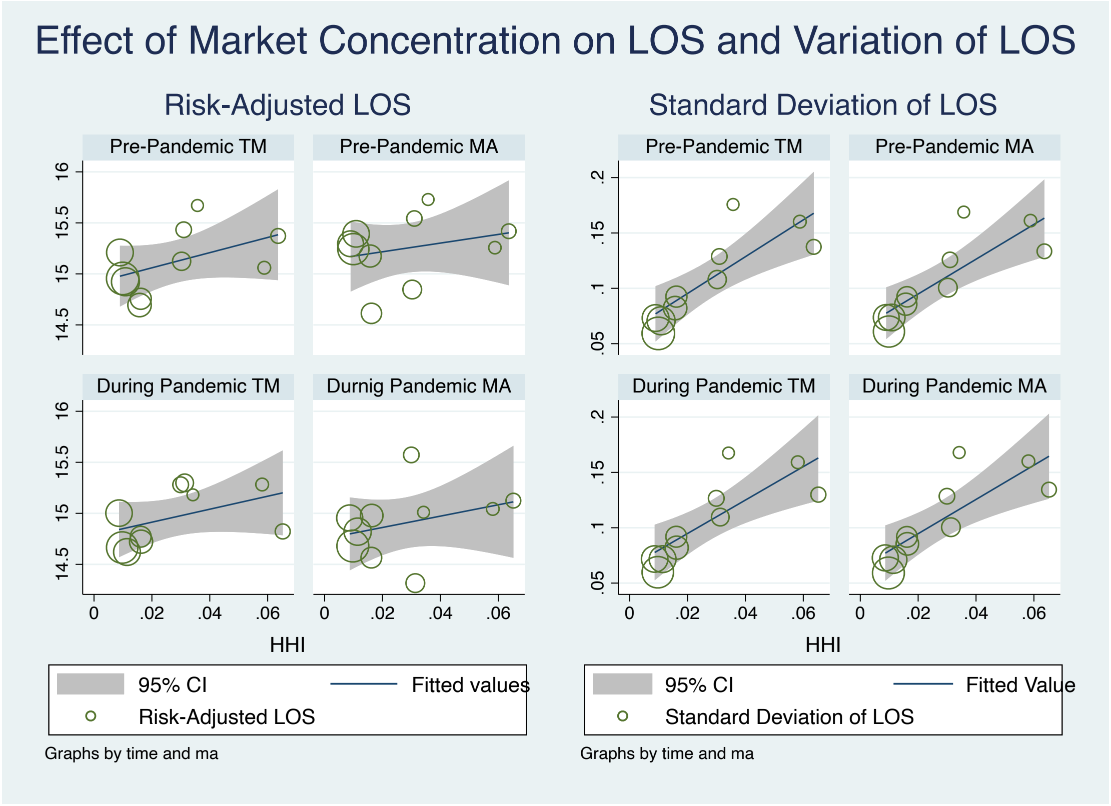

Source: Authors' Analysis of the Inpatient Rehabilitation Facility - Patient Assessment Instrument (IRF-PAI) data 2019-2020.

Notes: Acronyms: HHI- Herfindahl-Hirschman index, TM-Traditional Medicare; MA-Medicare Advantage; CI- Confidence Interval.

Appendix A6 (Figure). Association of MA Penetration with Risk-Adjusted Length of Stay (LOS) and Variation of LOS

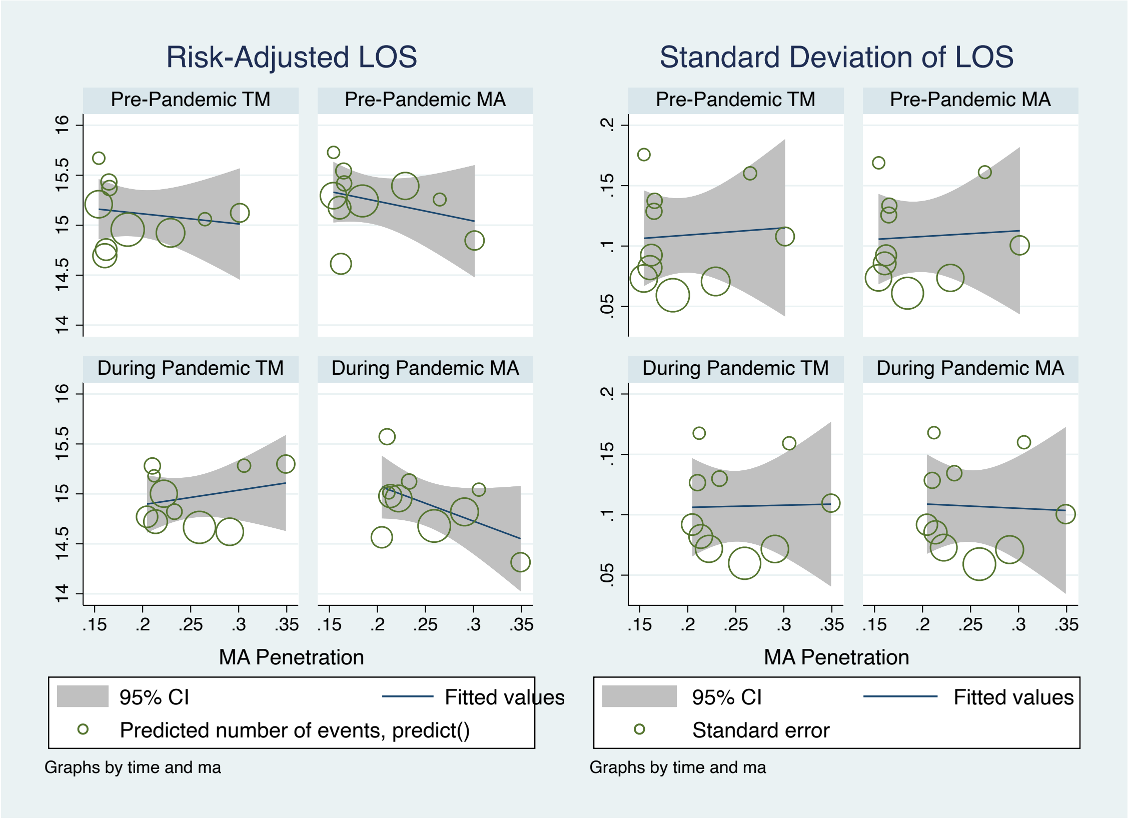

Source: Authors' Analysis of the Inpatient Rehabilitation Facility - Patient Assessment Instrument (IRF-PAI) data 2019-2020.

Notes: Acronyms: TM-Traditional Medicare; MA-Medicare Advantage; CI- Confidence Interval.
